# Exploring the influence of core-self evaluations, situational factors, and coping on nurse burnout: A cross-sectional survey study

**DOI:** 10.1371/journal.pone.0230883

**Published:** 2020-04-02

**Authors:** Nina Geuens, Helena Verheyen, Peter Vlerick, Peter Van Bogaert, Erik Franck

**Affiliations:** 1 Centre of Expertise The Cycle of Care, Karel de Grote University College, Antwerp, Belgium; 2 Department of Work, Organisation and Society, Ghent University, Ghent, Belgium; 3 Centre for Research and Innovation in Care, Department of Nursing and Midwifery Sciences, Faculty of Medicine and Health Sciences, University of Antwerp, Antwerp, Belgium; East Carolina University Brody School of Medicine, UNITED STATES

## Abstract

Stress has become an inherent aspect of the nursing profession. Chronically experienced work stress can lead to burnout. Although situational stressors show a significant influence on burnout, their power to predict the complete syndrome is rather limited. After all, stressors only exist “in the eye of the beholder”. This study aimed to explore how individual vulnerability factors such as core-self evaluations and coping, contribute to burnout in relation to situational stressors within a population of hospital nurses.

Cross-sectional data was collected in 2014, using five validated self-report instruments: Dutch Core Self Evaluations Scale, Nursing Work Index Revised, Utrecht Coping List, Ruminative Response Scale, and Utrecht Burnout Scale. 219 of the 250 questionnaires were returned.

Core-self evaluations, situational factors and coping each contributed significantly to the predictive capacity of the models of the separate burnout dimensions. Core-self evaluations was significantly related to emotional exhaustion. It was suggested that Core-self evaluations might be placed at the initiation of the loss cycle. However, further research is warranted.

## Introduction

Stress has become an inherent aspect of the nursing profession [1], due to the regular witnessing of pain and death, lack of decision latitude, role stress, low support levels of supervisors, interpersonal conflicts, and communication problems [2]. In the long term, chronically experienced work stress can generate the development of psychological strain or burnout [3]. According to Aiken et al. (2012) nurses across Europe and the USA are at risk for burn-out [4]. For instance, in Belgium 7–12% of nurses have a high risk of developing burnout and an additional 2–7% score above the diagnostic cut-off for burnout [5, 6].

The concept of burnout, as first described by Freudenberger in 1974, refers to a protracted course of distress in which one is unable to cope with stressors over an extended period of time, leading to depletion of the body’s defences and ultimately physical and emotional exhaustion [7] syndrome by describing three dimensions: 1) emotional exhaustion, which refers to feeling depleted of all resources and overwhelmed, emotionally speaking and experiencing extreme emotional and/or physical fatigue; 2) depersonalization, which entails tendencies to maintain distance between oneself and one’s work, expressed by treating patients as objects and assuming an indifferent and cynical attitude towards them; and 3) reduced personal accomplishment, which refers to feelings of incompetence and lack of personal achievement in the job [3, 8].

Van Bogaert et al. (2013) demonstrated that these three dimensions of burnout were influenced by situational stressors for instance the nurse-physician relationship, nurse management at the unit level and hospital management and organizational support [9]. Although, external stressors show a significant influence on the dimensions of burnout, their power to predict the complete psychological syndrome is rather limited. For instance, not every individual who is exposed to the same situational stressors will develop symptoms of burnout. This has led to the recognition that the individual vulnerability or diathesis is an additional predisposing factor for the development of burnout. After all, stressors only exist “in the eye of the beholder” [10]. This implies that all individuals have their own “turning point” at which they will develop burnout, a point that depends on the interaction between individual vulnerability and the amount of stressors experienced [11]. As such, a dysfunctional relation between the work environment and a person can result in burnout. Maslach (2003) confirmed this concept by stating that research should avoid an isolated focus on either the individual or the context [12, 13]. Therefore, this study strives for a more complete model for the development of burnout by expanding upon the validated model of Van Bogaert et al. (2013), which focusses on situational stressors, by adding several individual vulnerability factors which have previously been related to burnout.

One of these individual factors is core self-evaluations (CSE). Judge et al. (1997) combined four core traits with relatively large intercorrelations: 1) self-esteem, which is the degree to which you approve yourself and how successful, worthy, capable and significant you see yourself; 2) generalized self-efficacy, which covers the appraisal of how capable you perform across many contexts at a global level; 3) neuroticism, which represents emotional instability and belongs to the Big Five personality traits; and 4) locus of control, which refers to the assumption of being able to control the environment [14]. Judge et al. (1997) stated that core self-evaluations exist as a higher order factor overarching these core traits [15]. They suggested that CSE influence people’s self-appraisal as well as the appraisal of others and the world on a subconscious level, by bottom-line, fundamental evaluations of the world, others and themselves. Thus, the appraisal of specific situations, for instance work-evaluation, is affected by deeper and more fundamental self-appraisals, although most people are not aware that their CSE influences their behaviour or perceptions [14, 15].

Therefore, these CSE might influence the appraisal of situational stressors such as the nurse-physician relationship, nurse management at the unit level and hospital management and organizational support, and as a consequence indirectly influence the dimensions of burnout. Best et al. (2005) found that in healthcare employees in the South Texas Veterans Health Care System CSE may influence both job satisfaction and burnout [12]. Peng et al. (2016) confirmed these results in a population of Chinese nurses by describing that CSE indirectly affects job burnout, which was completely mediated by organizational commitment and job satisfaction [16]. Furthermore, van Doorn and Hülsheger [17] endorse this theory by suggesting that CSE functions as an active psychological buffer against the effects of job demands on strain reactions in a population of German hospital employees. However, it is argued by some authors that CSE might be culturally dependent as the role of self in motivation and cognition varies within cultures [14, 16, 18]. As such, it might be interesting to conduct similar studies in various countries.

CSE might thus indirectly determine whether specific internal and/or external demands are perceived as disturbing, exceeding or threatening a person’s resources. After appraising the stressor, the next step in the stress process is determining how to respond best to these stressors. This is where coping mechanisms come into play. Coping entails constantly adapting cognitive, behavioural, emotional and social efforts to manage the demands [10, 19] and will thus determine how someone reacts to these demands [20]. Researchers have suggested a wide variety of approaches for understanding coping strategies. Currently, the problem-focused, emotion-focused and avoidance dimensions are the three most cited approaches [10, 21, 22]. Problem-focused coping is a technique to diminish stress or strain by altering the person-environment relationship, through changing the environment or oneself, or by reducing the level of the stressor. Problem-focused coping is an active coping style that engages in behaviour which will solve the problem causing the distress, generating a plan of action or alternative solutions [10, 22]. Emotion-focused coping consists of direct efforts to reduce the stressful emotional reaction without attempting to affect the presence of the stressors. This coping strategy aims to acknowledge, understand, and express emotions and contains activities such as expressing sadness or anger, re-evaluation of the situation, wishful thinking, acceptance, accepting reassurance from friends, and focusing on your strengths [10, 22]. Avoidance coping entails efforts to distract yourself from and not think about the problem, denial, consuming alcohol or using drugs, or distancing or escaping from the situation that instigates the stress process [10].

Research on the relation between coping styles and burnout is manifold. In their meta-analysis Shin et al. (2014) described that problem-focused coping correlates negatively with the three burnout dimensions, whereas emotion-focused coping correlates positively with these dimensions. Additionally, they found that depersonalization and emotional exhaustion were closely related to emotion-focused coping, and reduced personal accomplishment was closely related to problem-focused coping [22]. Avoidance coping was considered maladaptive for chronic workplace stressors and in the long term can generate considerably higher levels of strain [23, 24]. Interestingly, Shin et al. (2014) also observed that the association between coping and burnout is much stronger among the nursing group than among a teacher or customer service worker group. Consequently, the stress mechanisms within the nursing population should be studied in more detail.

In addition, Kammeyer-Mueller et al. (2009) targeted the relationship between CSE, perceived stressors, coping and strain. The results of their meta-analysis on three of the CSE factors (emotional stability, self-esteem, locus of control) showed that individuals with negative CSE experience more stressors and more strain than individuals with positive CSE. In addition, individuals low in CSE practice more avoidance coping, somewhat more emotion-focused coping, and less problem-focused coping than individuals high in CSE.

In addition to the three coping strategies, a fourth strategy was included in the current study: ruminative coping. This term is primarily used to describe unintentional perseverative and repetitive thoughts in the absence of obvious external indications [25]. This coping strategy has been related to job strain, emotional exhaustion, and depressed mood [26–28]. After all, rumination may cause individuals to continue to reflect about their work, for instance work-related problems or superior’s demands, even after regular work hours, when they are not physically or deliberately performing work-related tasks, as such depleting their energy level. [28]. Furthermore, a dispositional ruminative style may result in paying additional attention to appraised stressors, increasing their importance and effect on the psychological wellbeing [26]. A distinction in ruminative style can be made between reflection and brooding. The first factor, reflection or reflective pondering, is described as “purposeful turning inward to engage in cognitive problem solving to alleviate one’s depressive symptoms”. Brooding, the second factor, reflects a passive comparison of some unachieved standards with one’s current situation. It has been shown that brooding, compared to reflection, represents the more maladaptive or detrimental subcomponent of rumination. However, reflection is also not entirely harmless [29].

In sum, extensive and mainly separated literature exists on situational and individual factors as well as potential burnout antecedents in various professional groups. However, so far a study combining situational stressors, CSE as a higher order factor, coping (including rumination) and demographical factors as risk factors for the separate burnout dimensions in a population of hospital nurses has not been executed before. Aiming to fill in this research void, our comprehensive study does not only add to the current literature but also may provide a deeper understanding of the development of burnout in the high risk population of nurses.

As such, this study aims to explore the relationship between CSE, situational factors, coping, and burnout within the nursing population. Based on previous research and developed models (see above), it is hypothesized that CSE, situational factors and coping style will each contribute to the dimensions of burnout: lower CSE will be related to higher burnout scores (H1); a more negative perception of situational stressors such as the nurse-physician relationship, nurse management at the unit level and hospital management and organizational support will be positively related to burnout (H2); and higher emotion-focused coping, avoidance coping, and brooding and lower problem-focused coping will be related to higher burnout scores (H3).

## Materials and methods

### Sample and participants

Building on previous research concerning CSE and burnout in a population of service providers, and coping and burnout in physicians, significant results were reported for respectively 265 and 139 respondents [18, 30]. Therefore, a minimum sample size of 200 respondents was predetermined. In previous studies conducted in these settings by the researchers a high response rate of 80% was achieved by providing a lottery scratch ticket as incentive. Therefore, 250 questionnaires were handed out.

Participants were recruited at random in 11 hospitals in the Dutch speaking part of Belgium. We aimed to include respondents from all different nursing specialty areas. Thirty-six units were selected through stratified random sampling in order to include this variety of unit types and thus obtain a broad spectrum of the nursing profession within the hospital setting. Three unit nurse managers refused to participate due to high workload. The unit nurse managers of the participating units were asked to choose a random day or shift and hand out questionnaires to all nurses working at that time. Depending on the number of nurses employed within the selected units, three to ten questionnaires were handed out per unit, with an average of seven. To avoid selection bias, the unit nurse managers were prompted to follow the selection procedure rigorously.

### Design and data collection

A cross-sectional research method was applied. Data collection was performed in February and March 2014 using a written (paper) questionnaire. The survey was announced by the unit nurse managers who handed the study information, informed consents and questionnaires to the sampled nurses. A form with instructions on how to complete and seal the questionnaire and informed consent form, the date of collection and arrival of the lottery scratch tickets was provided in the nursing station. After three weeks the completed and sealed questionnaires and informed consents were gathered by the researcher. The questionnaire consisted of five validated self-report instruments concerning core self-evaluations (Dutch Core Self Evaluations Scale), situational factors (Nursing Work Index Revised), Coping (Utrecht Coping List), rumination (Ruminative Response Scale), and burnout (Utrecht Burnout Scale), supplemented with several demographical questions and questions regarding job characteristics. No personal data such as name or address were collected. The usability of the questionnaire was evaluated by three independent nurse/researchers.

### Measures

#### Outcome variables

Burnout was evaluated using the Utrecht Burnout Scale (UBOS; Schaufeli & Van Dierendonck, 2000). This is the Dutch translation of the Maslach Burnout Inventory, which is based on 20 items of the MBI-Human Service Survey [31, 32], and measures the frequency of the main burnout symptoms on a seven-point scale ranging from 0 (never) to 6 (always). Belgian cut-off values were not available, therefore Dutch cut-off values specified for nurses were used to label high to very high levels of emotional exhaustion (mean score >2.12) and depersonalization (mean score >1.79 or >1.59 for men or women, respectively) and low to very low levels of personal accomplishment (mean score <3.57) [33]. In keeping with the guidelines, burnout was defined as having a high to very high score of emotional exhaustion and depersonalization combined with a low to very low score on personal accomplishment. Another term that is used in burnout research is “a high risk of burnout”. Respondents were identified as having a high risk when they experienced high to very high emotional exhaustion combined with either high to very high depersonalization or low to very low personal accomplishment [33]. In this study, Cronbach’s alpha for the dimension of emotional exhaustion totalled up to 0.89; 0.72 for the depersonalization dimension; and 0.79 for personal accomplishment.

#### Independent variables

First of all, a Dutch version of the **Core Self-Evaluations Scale** (NCSES) was included in the survey. The original scale was developed by Judge et al. (2003). It is a 12-item self-report instrument which calculates the score of the higher order construct (core self-evaluations) by combining the scores of four lower order constructs (self-esteem, generalized self-efficacy, neuroticism, and locus of control). Items such as “Overall, I am satisfied with myself”, “When I try, I generally succeed”, “There are times when things look pretty bleak and hopeless to me”, and “I determine what will happen in my life”, are rated from 1 (strongly disagree) to 5 (strongly agree) [34]. The scale sum score was calculated, after recoding some items. Cronbach’s alpha was 0.87.

Second, a validated and translated version of the **Nursing Work Index Revised** (NWI-R-vl) was added to the survey. This instrument consists of three dimensions or situational factors: nurse-physician relationship (3 items; e.g. “Physicians and nurses have good working relationships”), nurse management at the unit level (13 items; e.g. “Nurse managers consult with staff on daily problems and procedures”), and hospital management and organizational support (15 items; e.g. “Adequate support services allow nurses to spend time with patients”) [31, 35]. Respondents were asked to indicate the extent to which they agree with all these situational factors or statements in their current position on a 4-point Likert-type answer scale ranging from 1 (strongly disagree) to 4 (strongly agree). In the present study, Cronbach’s alpha's were respectively 0.87, 0.82, and 0.84.

Third, Coping strategies were measured using the **Utrecht Coping List** (UCL) [36] which is based on the classification of coping behaviour of Westbrook (1979) [20, 37]. It measures coping as a personality style, which does not conceptualizes coping as a fixed state, but it is assumed that individuals exhibit a certain preference of coping style within various situations. The instrument has 7 subscales representing 7 types of coping styles, including active coping, seeking social support, expressing emotions, reassuring or soothing thoughts, passive coping, palliative coping, and avoidance or waiting. Three of these coping strategies were selected to limit the size of the studied model, while still representing problem-focused, emotion-focused and avoidance coping. The first selected coping strategy is active coping (7 items), which is characterized by looking at the situation from all angles, analysing it and acting goal-oriented and with confidence to solve the problem (e.g. “Acting immediately when problems occur”). As such it can be classified as a problem-focused coping style. The second selected coping strategy is a type of emotion-focused coping (3 items) and involves expressing emotions, which entails showing annoyance or anger and letting off steam (e.g. “Expressing your annoyance”). The final selected coping strategy is a type of avoidance coping (8 items) and entails avoiding or waiting by letting the situation run its course, evading the situation or just watching to see what will happen (e.g. “Admitting to avoiding difficult situations”). Respondents had to indicate how often they thought or reacted in a certain way when confronted with problems or unpleasant circumstances on a 4-point Likert-type scale ranging from 1 (seldom or never) to 4 (very often). The three scale scores were calculated by adding the items related to each coping style. Dutch cut-off values based on a population of railway employees and nurses were used to label high to very high levels, average or low to very low levels of each coping style [20, 36]. In the current study, Cronbach’s alphas were respectively 0.82, 0.63, and 0.71.

Finally, the **Ruminative Response Scale** (RRS) was included in the survey and measured the ruminative thoughts and behaviours when feeling disheartened. This instrument, which was originally developed by Nolen-Hoeksema & Morrow (1991) and adapted by Treynor et al. (2003) consists of 22 items [38, 39]. However, we focused on a subset of 10 items, which can be equally divided over 2 subscales: Reflection (5 items); e.g. “I write down what I am thinking and analyse it”) and Brooding (5 items; “I think: ‘What am I doing to deserve this?’”). As such, the remaining 12 items can be discarded as they have too many similarities with depressive symptoms [40]. Respondents had to indicate how often they thought or reacted in a certain way when feeling sad, disheartened or depressed on a 4-point Likert-type scale ranging from 1 (almost never) to 4 (almost always). Scale sum scores were calculated. Cronbach’s alpha’s were respectively 0.79 and 0.83.

### Data analysis

Hierarchical regression analysis within IBM® SPSS Statistics V22.0 [41] was used to analyse the data. No missing values were allowed for the calculation of CSE, coping styles and rumination as these used sum scores. The Utrecht Burnout Scale allowed two missing values for the dimensions of emotional exhaustion and personal accomplishment, and one missing value for depersonalization [33]. For the descriptive analysis of the variables cut-off values were applied when available.

To examine whether the hypothesized relationship exists between CSE, situational factors, coping, and burnout, we conducted three hierarchical regression analyses (method enter) with the separate burnout dimensions as dependent variable (Tables 2–4). In the first block, age and gender were entered as control variables, as they have been known to influence burnout [42, 43]. In the second block, the higher order factor of CSE was entered. In the third block, the three situational factors of “nurse-physician relationship”, “nurse management at the unit level”, and “hospital management and organizational support” were added simultaneously. In the fourth and final block the five measured coping styles (problem-focused, emotion-focused, avoidance coping, brooding and reflection) were entered at once.

When the assumptions were checked, no multicollinearity was found using collinearity diagnostics between the variables in the regression analyses. Determinants of multicollinearity were considered a very low tolerance (< .10) and/or a high VIF (>10). Furthermore, no heteroscedasticity, extreme outliers or influential data points were observed for the regressions with emotional exhaustion, depersonalization, and personal accomplishment as dependent variables.

Additionally, effect sizes were calculated to measure the strength of the results. Cohen’s f^2^ was calculated for the hierarchical regression in Tables 2–4 with .02 suggesting a small effect, .15 a medium effect and .35 a large effect [44].

### Ethics approval and consent to participate

The Strengthening the Reporting of Observational Studies in Epidemiology (STROBE) statement for cross-sectional studies was adhered to in this manuscript [45].

Ethics committee approval was obtained from the Ethics Committee of Antwerp University Hospital and University of Antwerp Belgium designated as central committee (B300201318842) as well as approval from the local ethics committees of each participating hospital. Every eligible staff nurse was asked to fill out an informed consent form after reading the complete study information. To ensure privacy after completion a sealable envelope was provided together with the questionnaire and informed consent form. During data entry all data were anonymised by separating the questionnaires from the informed consent form.

## Results

Two hundred nineteen of the 250 questionnaires were recovered, resulting in a response rate of 88%. However, of these 219 questionnaires, 35% contained some missing data. As the allowed number of missing variables per (sub)scale was adhered to, not all analyses could be conducted on the total sample of 219. The available sample size, after exclusion of the missing data, is presented at the top of each table. The population consisted for 84% of female nurses with a mean age of 39 years (SD 10.9) and a mean of 16 years nursing experience (SD 10.8). Eighty-four percent of staff nurses had a working regime of 75% or more of a full-time position and most worked alternating 8-hour shifts (68%).

### Descriptive analysis of CSE, situational factors, coping styles, and burnout

The correlations between the dependent and independent variables and the means are displayed in Table 1. Nurses’ ratings of the situational factors of “nurse–physician relationship” and “nurse management at the unit level” were in the favourable range (> 2.5, suggesting agreement with the statements). “Hospital management and organizational support” was assessed as fairly reasonable.

**Table 1:** Mean, SD, Range, and correlations of the research variables.

	Mean	SD	Range	1	2	3	4	5	6	7	8	9	10	11
1. Emotional Exhaustion	1.5	0.9	0.0–4.9											
2. Depersonalization	1.1	0.7	0.0–4.6	.57**										
3. Personal Accomplishment	4.1	0.8	1.9–5.9	-.37**	-.50**									
4. CSE	45.1	7.2	24.0–60.0	-.55**	-.32**	.39**								
5. Nurse-Physician Relationship	2.8	0.6	1.0–4.0	-.31**	-.35**	.44**	.19*							
6. Nurse Management at the Unit Level	2.9	0.4	1.8–3.9	-.48**	-.44**	.44**	.28**	.41**						
7. Hospital Management and Organizational Support	2.4	0.4	1.2–3.3	-.46**	-.39**	.38**	.27**	.53**	.64**					
8. Problem Focused Coping	18.9	3.4	8.0–28.0	-.25**	-.23**	.38**	.47**	.16*	.19*	.23**				
9. Emotion Focused Coping	6.6	1.6	3.0–12.0	.34**	.26**	-.20**	-.25**	-.09	-.24**	-.23**	-.18**			
10. Avoidance Coping	16.4	3.2	9.0–25.0	.15*	.22**	-.22**	-.26**	.01	-.06	-.07	-.11	.12		
11. Reflection	7.2	2.4	5.0–18.0	.43**	.22**	-.05	-.42**	-.01	-.12	-.23**	-.13	.19**	.03	
12. Brooding	8.3	2.7	5.0–18.0	.51**	.33**	-.15*	-.55**	-.09	-.18*	-.22**	-.30**	.35**	.13	.66**

*p < 0.05

**p < 0.001

Concerning the coping styles 31% of nurses had a high to very high score on problem-focused or active coping and 15% a low to very low score. 48% of nurses scored high to very high on emotion-focused coping and 8% low to very low. Furthermore, 46% exhibited a high to very high score on avoidance coping and 4% low to very low.

Regarding the separate burnout dimensions, 22% of nurses experienced high to very high levels of emotional exhaustion, 22% had high to very high levels of depersonalization, and 25% scored low to very low on the dimension of personal accomplishment. This totalled up to 9% of nurses with a high risk for the development of burnout and an additional 7% scoring above the diagnostic cut-off for burnout.

### Hierarchical regression analysis

In order to examine the relationships between CSE, situational factors, coping, and burnout, hierarchical regression analyses were conducted (Tables 2–4).

**Table 2:** Hierarchical regression analysis of emotional exhaustion (N = 143).

BLOCK	Variables	B	Standard error	β	t	p	CI	R^2^ Change (p)
**1**	**Gender**^**a**^	-.31	.15	-.13	-2.10	.04	-.60 –-.02	.04 (.07)
	**Age**	-.00	.01	-.02	-0.40	.69	-.01 –.01	
**2**	**CSE**	-.03	.01	-.23	-2.80	.01	-.05 –-.01	.31 (< .001)
**3**	**Nurse-Physician Relationship**	-.13	.11	-.08	-1.17	.25	-.36 –.09	.13 (< .001)
	**Nurse Management at the Unit Level**	-.51	.20	-.21	-2.61	.01	-.90 –-.12	
	**Hospital Management and Organizational Support**	-.26	.20	-.11	-1.29	.20	-.66 –.14	
**4**	**Problem Focused Coping**	-.01	.02	-.03	-0.38	.71	-.05 –.03	.08 (< .001)
	**Emotion Focused Coping**	.04	.04	.07	1.11	.27	-.03 –.11	
	**Avoidance Coping**	.01	.02	.04	0.64	.52	-.02 –.05	
	**Reflection**	.05	.03	.12	1.39	.17	-.02 –.11	
	**Brooding**	.07	.03	.22	2.23	.03	.01 –.14	

linear regression analysis, enter method; CI = 95% confidence interval; p = p-value; Adjusted R^2^ = .52; effect size f^2^ = 1.09 (.02 = small, .15 = medium, .35 = large)

^a^ = women vs men

**Table 3:** Hierarchical regression analysis of depersonalization (N = 142).

BLOCK	Variables	B	Standard error	β	t	p	CI	R^2^ Change (p)
**1**	**Gender**^**a**^	-.53	.13	-.26	-3.93	< .001	-.79 –-.63	.17 (< .001)
	**Age**	-.02	.00	-.28	-4.40	< .001	-.03 –-.01	
**2**	**CSE**	.01	.01	.07	0.83	.41	-.01 –.03	.09 (< .001)
**3**	**Nurse-Physician Relationship**	-.28	.10	-.21	-2.72	.01	-.49 –-.08	.16 (< .001)
	**Nurse Management at the Unit Level**	-.47	.18	-.22	-2.64	.01	-.83 –-.12	
	**Hospital Management and Organizational Support**	-.11	.19	-.05	-0.58	.56	-.48 –.26	
**4**	**Problem Focused Coping**	-.03	.02	-.12	-1.69	.09	-.07 –.01	.08 (.001)
	**Emotion Focused Coping**	.00	.03	-.00	-0.01	.99	-.06 –.06	
	**Avoidance Coping**	.03	.02	.12	1.95	.05	-.001- .06	
	**Reflection**	.02	.03	.05	0.58	.56	-.04 –.08	
	**Brooding**	.07	.03	.26	2.46	.02	.01 –.13	

linear regression analysis, enter method; CI = 95% confidence interval; p = p-value; Adjusted R^2^ = .47; effect size f^2^ = . 88 (.02 = small, .15 = medium, .35 = large)

^a^ = women vs men

**Table 4:** Hierarchical regression analysis of personal accomplishment (N = 143).

BLOCK	Variables	B	Standard error	β	t	p	CI	R^2^ Change (p)
**1**	**Gender**^**a**^	-.01	.14	-.01	-0.08	.93	-.28 –.26	.04 (.07)
	**Age**	.01	.01	.15	2.18	.03	.001 –.02	
**2**	**CSE**	.02	.01	.17	1.81	.07	-.002 –.04	.10 (< .001)
**3**	**Nurse-Physician Relationship**	.45	.11	.34	4.22	< .001	.24 –.66	.23 (< .001)
	**Nurse Management at the Unit Level**	.38	.18	.19	2.07	.04	.02 –.75	
	**Hospital Management and Organizational Support**	.14	.19	.07	0.71	.48	-.24 –.51	
**4**	**Problem Focused Coping**	.04	.02	.18	2.32	.02	.01 –.08	.07 (.01)
	**Emotion Focused Coping**	0.04	.03	-.08	-1.12	.26	-.10 –.03	
	**Avoidance Coping**	-.03	.02	-.14	-2.04	.04	-.07 –-.001	
	**Reflection**	.001	.03	.01	0.05	.96	-.06 –.06	
	**Brooding**	.04	.03	.16	1.41	.16	-.02 –.11	

linear regression analysis, enter method; CI = 95% confidence interval; p = p-value; Adjusted R^2^ = .39; effect size f^2^ = .64 (.02 = small, .15 = medium, .35 = large)

^a^ = women vs men

Determining factors for emotional exhaustion were gender, CSE, “nurse management at unit level”, and brooding (Table 2). The complete model explained 52% of the variance in emotional exhaustion. Age and gender explained 4% of this variance in block 1 (R^2^ = .04; F = 2.69; p = .07). CSE explained an additional 31% in block 2 (R^2^ Change = .31; F Change = 65.24; p < .001). In block 3 the situational factors explained 13% (R^2^ Change = .13; F Change = 11.46; p < .001) and finally the coping styles added 8% (R^2^ Change = .08; F Change = 4.79; p < .001) to the explained variance in block 4 of the regression.

For the burnout dimension of depersonalization, a significant impact of gender, age, “nurse-physician relationship”, “nurse management at the unit level”, avoidance coping, and brooding was found (Table 3). This model explained 47% of the variance in depersonalization. Gender and age explained 17% of this variance in block 1 (R^2^ = .17; F = 14.66; p < .001). CSE increased the model’s predictive capacity with 9% in block 2 (R^2^ Change = .09; F Change = 16.67; p < .001). The situational factors added 16% in block 3 (R^2^ Change = .16; F Change = 12.93; p < .001). Lastly, the coping styles explained an additional 8% (R^2^ Change = .08; F Change = 4.34; p = .001) in the fourth block.

Age, “nurse-physician relationship”, “nurse management at the unit level”, problem focused coping, and avoidance coping were found to be determining factors for the burnout dimension of personal accomplishment (Table 4). This model explained 39% of the variance in personal accomplishment. Control variables (age and gender) explained 4% of this variance in block 1 (R^2^ = .04; F = 2.68; p = .07). CSE explained an extra 10% in block 2 (R^2^ Change = .10; F Change = 16.78; p < .001). The situational factors explained 23% of the variance in block 3 (R^2^ Change = .23; F Change = 16.66; p < .001), and finally, the coping styles added 7% (R^2^ Change = .07; F Change = 3.03; p = .01) to the predictive capacity in block 4 of the regression.

## Discussion

This study aimed to explore the relationship between burnout and three types of potential antecedents (CSE, situational factors and coping) within a sample of hospital nurses.

Results show that each of the three types of burnout antecedents contributed statistically significant to the prediction of at least one of the three burnout dimensions.

Furthermore, the predictive capacity of the regression models proved to be substantial with large effect sizes and explained variances reaching up to 52%, 47% and 39% for emotional exhaustion, depersonalization and personal accomplishment respectively. Taking into account that causes for burnout are multifactorial and that only a limited amount of predictors were studied, this proportion of explained variance is very meaningful.

Regarding the demographical variables, relationships were found for gender and age. In line with previous research concerning personality, interpersonal behaviour and burnout in a nursing population, the current study found that female nurses experienced less emotional exhaustion and depersonalization than male nurses [5]. However, the finding that women are less likely to report emotional exhaustion is contradicted in the meta-analysis of Purvanova & Muros (2010). The increased risk for male nurses that was found in the study at hand might be explained by the fact that domination of an occupation by one gender is likely to create less positive experiences for members of the underrepresented gender [43]. Men in female-typed occupations often face emotional and interpersonal challenges that they are not well-prepared for, as these skills are not mandatory for successful realisation of the male gender role, thus decreasing the perception of internal resources to cope with these job demands. Moreover, men in healthcare are more often subjected to adverse social behaviour, including intimidation at work and acts of physical and verbal violence [46], as such increasing the job demands [5].

Furthermore, age was positively related to personal accomplishment and negatively to depersonalization. These results are in line with previous research which states that compared to their older counterparts, nurses under 30 years of age are more likely to experience feelings of agitation and less likely to engage in techniques to manage these feelings. Younger nurses also reported significantly higher rates of burnout and this was particularly true among those experiencing higher levels of agitation at work [47, 48]. However, the negative relation between age and emotional exhaustion could not be confirmed in the study at hand.

Concerning the three hypotheses on the relationship between the burnout dimensions and CSE (H1), situational factors (H2) and coping (H3), an explanation for the established results can be provided based on the job demands and job resources model. Different scholars have shown that burnout and engagement experienced at work result from the combination of two sets of working conditions, i.e., job demands, and the job resources available to cope with these demands following two underlying psychological processes: an energy-draining process and a motivational process, respectively [49, 50]. Unbalanced job demands and job resources were identified as part of a loss cycle or strain process. Expansions of job resources, on the other hand, were found to predict work engagement in a gain cycle or motivational process [51–55]. In this article we focused on the loss cycle as this study measured burnout, not engagement. After all, engagement can be described as an independent and distinct (albeit related) concept [51, 56].

Next, a statistically negative and strong relation between CSE and emotional exhaustion was found. This is consistent with previous research [12, 16, 18]. Additionally, in line with earlier findings the situational factor of “nurse management at unit level” remained consistently significant in the regression models of the three burnout dimensions [9]. Interestingly, the impact of hospital management and organizational support on personal accomplishment was not confirmed in our study results. “Nurse-physician relationship” contributed significantly to the models of personal accomplishment and depersonalization, the latter as previously reported [9].

Concerning the loss cycle, low CSE could instigate the perception of unfavourable work demands and few internal resources to deal with these demands. Consequently, this misfit or imbalance between job resources and job demands could possibly initiate the vicious loss circle, lowering CSE, perception of situational factors and adequate coping skills even further and ultimately causing burnout. Best et al. (2005) suggest that healthcare employees perceiving a constraining work environment and low on CSE are at risk for burning out. Additionally, they indicate that employees who are fundamentally lower in generalized self-efficacy, self-esteem, emotional stability (opposite of neuroticism) and who have an external locus of control may be more likely to perceive greater constraints in the organizational environment than their high CSE colleagues [12]. Moreover, Llorens-Gumbau & Salanova-Soria (2014) confirm in their longitudinal study of teachers that the energy depletion process starts with the perception of a high number of obstacles that lead to high levels of cynicism and exhaustion and consequently decreases self-efficacy–one of the four factors of CSE–even more [50]. Additionally, Maslach and colleagues propose on the long-standing notion that burnout results from a misfit between the individual and the job. The greater the perceived mismatch is within the six areas of work life, the greater the likelihood of burnout. These areas of work life encompass the extent to which someone experiences control, workload, reward, fairness, community and values congruence [51, 57]. The essential role of this misfit was confirmed in a recent qualitative study which described that the essence of the development of nurse burnout was found in the discrepancy between the vulnerability factors and situational stressors. The individual or vulnerability factor of “being passionate about doing well or being good” on its own did not cause substantial stress or burnout. It was the discrepancy between this factor and the external stressors of “teamwork”, “manager”, and “work and private circumstances” that led to feelings of stress and burnout [58].

However, the influence of perception through CSE could not be demonstrated for the two remaining burnout dimensions (personal accomplishment and depersonalization) as CSE did not maintain its statistical significance in the models after the other independent variables were added. CSE was significant when entered in block 2 of the regression models of depersonalization and personal accomplishment. Yet, in block 4 they proved no longer significant. These findings might indicate that CSE can be placed at the initiation of the loss cycle. After all, various researchers confirmed a directional relationship among the three-burnout dimensions: emotional exhaustion predicts depersonalization, which predicts reduced personal accomplishment [51, 59, 60]. This directionality was also portrayed in the model of Van Bogaert et al. (2013) which this study aimed to expand upon [9]. Therefore, it can be suggested that CSE directly influences the development of emotional exhaustion, and as a consequence exerts an indirect influence on the advancement to depersonalization and ultimately to feelings of reduced personal accomplishment. After all, despite CSE not maintaining its significance in the models of depersonalization and personal accomplishment, several situational factors and coping styles did remain in these models.

With regard to the coping styles, brooding proved to be positively related to depersonalization and emotional exhaustion; avoidance coping negatively to personal accomplishment and positively to depersonalization; and problem-focused coping was positively related to personal accomplishment. Donahue et al. (2012) confirmed that brooding reduces the amount of recovery experiences, resulting in negative outcomes such as sleep problems, health complaints, fatigue or even emotional exhaustion. Furthermore, Shin et al. (2014) confirmed that reduced personal accomplishment is closely related to problem-focused coping and displays a negative relation to planning, problem solving, and active coping [22, 61]. However, in contradiction to the meta-analysis of Shin et al. (2014), our study results were unable to confirm the close relation between emotion-focused coping and depersonalization [22].

Based on the theory of the loss cycle, we suggest that low CSE might indirectly influence the burnout dimensions through their negative impact on the perception of situational factors, which in turn may lead to more avoidance and rumination coping and less problem-focused coping and ultimately increase the dimensions of burnout. This additional hypothesis on the mechanism and the order in which the factors influence each other can be explored in more detail for the main burnout dimension (cf. emotional exhaustion). Determining factors for emotional exhaustion were gender, CSE, “nurse management at unit level”, and brooding. This may imply that CSE influences the perception of the nurse management, which in turn increases brooding about the nurse management, resulting in emotional exhaustion of the individual nurse. This hypothesis is substantiated further in Table 1 which shows that CSE is positively correlated to “nurse management at the unit level”. Therefore, as CSE is lower, the perception of the nurse management is worse. After all, a fundamental self-appraisal based on low self-esteem, self-efficacy, locus of control and high neuroticism can lead to perceiving the direct care context and front-line leadership as unfavourable and feeling unable or even powerless to do anything about it. Additionally, “nurse management at the unit level” had a negative correlation to brooding, implying that as the perception of the nurse management becomes more negative, the level of brooding increases. Work often constitutes an important part of the life and identity of individuals. Therefore, when nurses are dissatisfied with the nursing management at unit level they might continue to ponder about these work related problems or the behaviour of their supervisor even after working hours [28]. Additionally, brooding displayed a strong positive relation to emotional exhaustion, indicating that more brooding is related to more emotional exhaustion.

However, these speculations could not be tested in the study at hand due to its cross-sectional research design. Further research is warranted to confirm the causal relationship between these factors.

### Limitations, strengths and future research

Burnout research, including the current study, reveals the complexity of the concept, and places the personal prolonged stress experience within a broader organizational context of people’s relation to their work [51, 62]. This study provides several important factors in the development of burnout, such as CSE and coping mechanism, relevant for the practice community, to understand and make more distinctions between personal vulnerability for stress and burnout and inadequate work place conditions in order to develop burnout prevention strategies accurately [63, 64].

Because of the cross-sectional methodology verification of causal relationships or directionality between the different factors were not established. Therefore, we would like to invite further research to determine whether our results can be confirmed or extended. A longitudinal study including path-analysis may be interesting as to investigate these interactions between CSE, situational factors, coping and burnout. Furthermore, qualitative studies could explore the relationship between these factors more into depth. Furthermore, because 35% of returned questionnaires contained some missing data, a relatively small sample size was suitable for hierarchical regression modelling. Therefore, additional research in a larger sample might be desirable. In addition, the possibility of non-response error in the study at hand must be recognized. Additionally, burnout was calculated based on the scores of a self-report instrument. This could render our results vulnerable to common-method variance as participants might not have an accurate perception of their actual coping style or might feel pressured to provide socially desirable answers concerning CSE or situational factors. Moreover, denial of the problem has been described as a symptom of burnout [65], which causes underreported symptoms of burnout. Objective clinical diagnosis of burnout by a professional may delete this bias. However, it has also been argued that for studying individual factors, self-reports might be the most valid measuring method, as the participants are the most suitable party to report their own perception and level of burnout [66].

### Relevance to clinical practice

Although the current study was unable to confirm directionality between the factors, it could be established that both individual factors and situational stressors contributed to each of the burnout dimensions. Therefore, we suggest that burnout interventions are aimed at both factors to achieve the best results. This is confirmed by Awa, Plaumann & Walter [67] who found in their review of burnout intervention programs that person-directed interventions reduced burnout within 6 months or less, while a combination of both person- and organization-directed interventions showed longer lasting positive effects of 12 months and more. Supplementary, these interventions could utilize the loss and gain cycles of the job demands-job resources theory as a guideline to gain inside in the mechanism of the development of burnout.

## Conclusion

This study aimed to explore the relationship between three potential antecedents (CSE, situational factors and coping) of burnout in hospital nurses. Altogether, the multivariate results demonstrated that hospital nurses' burnout is statistically significant related to [1] demographical variables (e.g age and gender); external/situational stressors (e.g. the “nurse management at unit level” and “nurse-physician relationship”) and [3] to internal personal characteristics such as coping style (i.e. avoidance coping, brooding and problem focussed coping) and core self-evaluations.

Moreover, we found that burnout antecedents seem to be partly burnout dimension specific. For instance, we remarkably showed that CSE was related only to the core burnout dimension emotional exhaustion and not meaningfully to the two other burnout dimensions (personal accomplishment and depersonalization).

Applying the job demands-job resources theory of the strain process or loss cycle, this latter result suggest that CSE can be placed at the initiation of the loss cycle. Therefore, CSE may influence the development of emotional exhaustion directly, and as a consequence exert an indirect influence on the advancement to depersonalization and ultimately to feelings of reduced personal accomplishment. However, further research on the potential direct and/or indirect effect of CSE on the development of burnout is recommended.
